# Expression of acid-sensing ion channels and selection of reference genes in mouse and naked mole rat

**DOI:** 10.1186/s13041-016-0279-2

**Published:** 2016-12-13

**Authors:** Laura-Nadine Schuhmacher, Ewan St. John Smith

**Affiliations:** 1 Department of Pharmacology, University of Cambridge, Tennis Court Road, Cambridge, CB2 1PD UK; 2 Department of Cell & Developmental Biology, University College London, Gower Street, London, WC1E 6BT UK

**Keywords:** Acid-sensing ion channel, Gene expression, geNorm, Normalization, Quantitative real-time PCR, Naked mole-rat, Housekeeping gene selection, Central nervous system, Comparative study

## Abstract

Acid-sensing ion channels (ASICs) are a family of ion channels comprised of six subunits encoded by four genes and they are expressed throughout the peripheral and central nervous systems. ASICs have been implicated in a wide range of physiological and pathophysiological processes: pain, breathing, synaptic plasticity and excitotoxicity. Unlike mice and humans, naked mole-rats do not perceive acid as a noxious stimulus, even though their sensory neurons express functional ASICs, likely an adaptation to living in a hypercapnic subterranean environment. Previous studies of ASIC expression in the mammalian nervous system have often not examined all subunits, or have failed to adequately quantify expression between tissues; to date there has been no attempt to determine ASIC expression in the central nervous system of the naked mole-rat. Here we perform a geNorm study to identify reliable housekeeping genes in both mouse and naked mole-rat and then use quantitative real-time PCR to estimate the relative amounts of *ASIC* transcripts in different tissues of both species. We identify *RPL13A* (ribosomal protein L13A) and *CANX* (calnexin), and *β-ACTIN* and *EIF4A* (eukaryotic initiation factor 4a) as being the most stably expressed housekeeping genes in mouse and naked mole-rat, respectively. In both species, ASIC3 was most highly expressed in dorsal root ganglia (DRG), and ASIC1a, ASIC2b and ASIC3 were more highly expressed across all brain regions compared to the other subunits. We also show that ASIC4, a proton-insensitive subunit of relatively unknown function, was highly expressed in all mouse tissues apart from DRG and hippocampus, but was by contrast the lowliest expressed ASIC in all naked mole-rat tissues.

## Introduction

Acid-sensing ion channels (ASICs) are a group of six ion channels encoded by four different genes, splice variants of the genes encoding ASIC1 and ASIC2 produce ASIC1a and ASIC1b, and ASIC2a and ASIC2b respectively [1]. The predominant endogenous activator of ASICs is protons and combinations of different ASIC subunits produces homo- and heterotrimers with different biophysical and pharmacological properties [2–4]. Thus the sensitivity and response to protons of a particular neuron depends upon the combination of ASICs that are expressed. Presynaptic stimulation has been shown to produce changes in extracellular pH through release of acidic synaptic vesicles into the synaptic cleft and thus protons may act as a neurotransmitters, with ASIC1a expressed in the postsynaptic membrane contributing to synaptic plasticity [5]. ASICs therefore have the potential to contribute to a variety of physiological and pathophysiological processes in the brain and evidence exists to support their involvement in: addiction [6], anxiety/fear [7–9], regulation of breathing [10], learning and memory [11] and excitotoxicity [12]. In the peripheral nervous system, ASICs are expressed by sensory neurons of the dorsal root ganglia (DRG) [13–16] and are involved in nociception and mechanosensation [17, 18]. Interestingly, the naked mole-rat (*Heterocephalus glaber*) shows no nocifensive response to acid due to a lack of activation of cutaneous sensory neurons [19], even though DRG neurons have ASIC-like currents and cloned ASIC1a and ASIC1b, as well as TRPV1, have biophysical properties that are indistinguishable from those of mice [14, 20]. Behavioral acid insensitivity is accounted for by a genetic variation in the voltage-gated Na^+^ channel subunit 1.7 (NaV1.7), which confers enhanced acid inhibition [14]. The NaV1.7 variation is conserved in a variety of hibernating species [21] and insensitivity to acid in these species is likely due to an adaptation to living in a hypercapnic environment [14, 22]. Considering that NaV1.7 expression is largely restricted to the peripheral nervous system [23, 24], any central adaptations to hypercapnia may result from differential ASIC expression and thus it is necessary to determine if there are any differences in ASIC brain expression in the naked mole-rat compared to mouse.

ASIC expression has been investigated using a variety of methods. *ASIC1* and *ASIC2* been found to be predominantly expressed in the brain by in situ hybridization, with a high level of mRNA expression in the cerebellum, hippocampus, habenula, amygdala and olfactory bulb [25–27]. This *ASIC1* expression pattern was recapitulated by immunohistochemistry and northern blot analysis of human *ASIC1* and *ASIC2* showed transcripts in the amygdala, hippocampus, thalamus, substantia nigra, subthalamic nuclei and caudate nucleus [7]. Using probes that differentiated between *ASIC1a* and *ASIC1b*, it has been shown that whereas *ASIC1a* is expressed in rat DRG, spinal cord and brain, *ASIC1b* expression is restricted to the DRG [28]. However, reverse-transcriptase PCR (RT-PCR) analysis showed that *ASIC1b*, along with all other *ASIC* transcripts, is expressed in mouse anterior pituitary tissue [29] and rat trigeminal mesencephalic nucleus neurons [30], suggesting that *ASIC1b* is present in certain brain regions. A variety of techniques have been used to demonstrate that both *ASIC2a* and *ASIC2b* show diffuse expression throughout the brain in a number of species, as well as the spinal cord and DRG [26, 31–33]. By contrast, initial work using northern blot and in situ hybridization suggested that in rats *ASIC3* was only present in DRG and was absent from the brain [34]. However, later work using a combination of western blot and RT-PCR showed that it was present in hippocampus, amygdala, caudate putamen, prefrontal cortex and hypothalamus, as well as and DRG [35]. Initial studies examining the expression of *ASIC4* using dot blot, northern blot and RT-PCR showed that it was present in the brain, spinal cord and DRG [36, 37], work that has been supported by more recent investigation using *ASIC4* transgenic marker mice [8]. By contrast with mice and rats, there is very little known about *ASIC* expression in naked mole-rats, although one study used RT-PCR to demonstrate that transcripts for *ASIC1a*, *ASIC1b*, *ASIC2a*, *ASIC2b* and *ASIC3* are present in DRG, but expression of *ASIC4* was not determined [14].

Overall, reports of *ASIC* expression lack consistency of methodology, both in selection and comparison of tissue and the method used to report quantities. To overcome these shortcomings, we developed a rigorous qPCR protocol using appropriate normalization and a large number of different tissues. RNA was extracted from mouse and naked mole-rat brain (olfactory bulbs, cerebellum, brain stem, cerebral cortex and hippocampus), spinal cord and DRG and cDNA produced. A species-specific geNorm assay was then used to find reliable housekeeping genes, which enabled a reliable and comparable estimate of the relative amounts of *ASIC* transcripts present.

## Methods

### Animals

All experiments were conducted in accordance with the United Kingdom Animal (Scientific Procedures) Act 1986 under a Project License (70/7705) granted to E. St. J. S. by the Home Office; the University of Cambridge Animal Welfare Ethical Review Body also approved procedures. Young adult animals were used in this study: male and female, 7-week-old C57/bl6 mice and 6-month old male and female naked mole-rats; considering the ~30 year life span of naked mole-rats, 6-month old animals can be considered young adults like the 7-week old mice. Mice were housed in groups of up to five mice per cage with nesting material and a red plastic shelter; the holding room was temperature controlled (21 °C) and mice were on a standard 12-h light/dark cycle with food and water available ad libitum. Naked mole-rats were bred in house and maintained in a custom-made caging system with conventional mouse/rat cages connected by different lengths of tunnel. Bedding and nesting material were provided along with a running wheel. The room was warmed to 28 °C, with a heat cable to provide extra warmth running under 2–3 cages, and red lighting (08:00–16:00) was used.

### RNA extraction and cDNA synthesis

Animals were decapitated and tissues were rapidly dissected under a sterile hood using sterilized dissection tools and immediately transferred to TRI reagent (Sigma, 1 ml for 100 mg tissue) in 1.5 ml tubes. Samples were stored at -80 °C until further use. Once fully defrosted, tissues were homogenized using Eppendorf micropestles and incubated for 5 min at room temperature (RT). After adding 1:10 volume of 1-bromo-3-chloropropane and shaking for 15 s, samples were incubated another 3 min at RT and then centrifuged at 10,000 rpm for 15 min at 4°C to separate the mixture into three phases: a red organic phase containing protein, an interphase containing DNA and a colorless aqueous phase containing RNA. The aqueous phase was transferred to a new 1.5 ml tube and 500 μl 100% ethanol were added. The mixture was then applied to a Zymo RNA Clean & Concentrator column and an in-column DNaseI digest and clean-up of RNA were performed according to kit instructions. The RNA was eluted in 20 μl and a 3 μl aliquot was taken before storing at -80 °C. To test the quality of the extracted RNA, 1.5 μl of the aliquot were used to determine the concentration, while the remaining 1.5 μl were used for gel electrophoresis. To test the integrity and quality of RNA, 500 ng samples were run on a 1% agarose gels with TAE buffer, RNA quality assessed by identification of the 28S, 18S and 5S ribosomal RNA bands on a 1% agarose gel; in the case of naked mole-rat RNA, one additional band is expected resulting from the split 28S subunit [38]. In addition, samples were measured using RNA spectroscopy (NanoVueTM, VWR). RNA was transcribed into cDNA using random nonamer and dT oligomer priming with the Precision nanoScript 2 kit (Primerdesign, for qPCR) according to the manufacturer’s directions.

### Primer design

Naked mole-rat ASIC and mouse ASIC1b primers were designed with the help of the PrimerQuest online tool (www.idtdna.com/PrimerQuest/) with the specification that the melting temperature be 57 °C, the GC content 45–55% and the product length 100–200 bp. Housekeeping primers and all other mouse ASIC primers were designed and tested by Primerdesign adhering to the same specifications Tables 1, 2 and 3). Primers were first tested by performing a standard RT-PCR using DreamTaq polymerase, each 20μl reaction containing 1X DreamTaq reaction buffer, 200 μM of dATP, dTTP, dGTP and dCTP, 0.5 μM of each primer, 10 ng cDNA and 0.02 U/μl DreamTaq DNA Polymerase and the PCR was run with the conditions 95 °C 5 min, 40X (95 °C 30 s, 60 °C 30 s, 72 °C 15 s), 72 °C 5 min, cool to 12 °C. The resulting bands were visualized on a 1% agarose gel to test primer specificity. Primers with only one product were deemed specific and were then tested in a qPCR reaction. For this validation, a standard curve experiment with RNA amounts of 25 ng, 12.5 ng, 5 ng, 2.5 ng, 1.25 ng and 0.5 ng was performed and a linear regression calculated for each primer pair. From the slope of this regression, the amplification factor (AF) and efficiency (E) could be calculated using the formula: AF = 10^-(1/slope)^ and E = AF − 1 × 100. Only primers with an efficiency of 70–130% were used in subsequent experiments.

**Table 1:** geNorm primer binding locations

Species	Gene	Accession no.	Anchor nucleotide	Sequence length (bp)
Mouse	*18S*	NR_003278.3	134	99
	*ACTB*	NM_007393.3	597	94
	*ATP5b*	NM_016774.3	1115	142
	*B2M*	NM_009735.3	202	159
	*CANX*	NM_007597.3	2827	127
	*CYC1*	NM_025567.2	514	203
	*EIF4A2*	NM_013506.2	876	215
	*GAPDH*	NM_008084.2	793	180
	*RPL13A*	NM_009438.5	691	180
	*SDHA*	NM_023281.1	2018	181
	*UBC*	NM_019639.4	2225	178
	*YWHAZ*	NM_011740.3	1045	195
Naked mole-rat	*ACTB*	XM_004840381	1231	143
	*B2M*	XM_013078376	128	116
	*CANX*	XM_013077439	1068	139
	*EIF4A2*	XM_004834818	404	145
	*GAPDH*	XM_004869398	455	118
	*GPI*	NM_001310272	1746	101
	*MDH1*	XM_004849985	548	112
	*RPL13A*	XM_004866919	362	92
	*SDHA*	XM_004845101	784	179
	*TOP1*	XM_004874029	128	116
	*YWHAZ*	XM_004850171	926	79

Table of specifications for primer binding according to Primerdesign, showing the sequence length in base pairs, the anchor nucleotide (central to the binding region) and accession number of the gene

**Table 2:** Mouse qPCR primers

Gene	Accession no.	Primer fw	Primer rv	Sequence length (bp)
*ASIC1a*	NM_009597	gaactgaagaccgaggaggag	gccgctcataggagaagatgt	112
*ASIC1b*	NM_001289791	tcagctaccctgacttgctcta	gagcggttgtagaaacgatgga	139
*ASIC2a*	NM_001034013	cgatggacctcaaggagagc	atacacgaagatgtggcggat	107
*ASIC2b*	NM_007384	cttgctgttgtcctggtcct	ttgttgttgcacacggtgac	123
*ASIC3*	NM_183000	ttcacctgtcttggctcctc	tgactggggatgggatttctaag	126
*ASIC4*	NM_183022	caccttgctggagatccttga	gtccgcagtggggtcttg	150

Forward (fw) and reverse (rv) primers in 5′-3′ orientation, accession number and length of product in base pairs

**Table 3:** Naked mole-rat qPCR primers

Gene	Accession no.	Primer fw	Primer rv	Sequence length (bp)
*ASIC1a*	XM_013078965.1	atgagataccagacacgcagat	gcagcatgtctcgaatgtcatg	144
*ASIC1b*	NM_001279840	ggtgccagtcatgtctttgtg	catgcgggtagctgaggtaata	136
*ASIC2a*	XM_013067767	gcacgttaccaaggtggatgag	tggtggtgagcctggagaa	101
*ASIC2b*	XM_004870614.2	tcgaaccgcctgctgtact	gggttgttattgcacacggtga	107
*ASIC3*	00000022115	atccgagtgcagatccacag	gttcctcaaagtcggagtccat	172
*ASIC4*	XM_004864511.1	ccagcaacttctctgtggtctat	actcctcctgctggatgtcta	163

Forward (fw) and reverse (rv) primers in 5′-3′ orientation, accession number and length of product in base pairs

### qPCR

For all qPCR experiments, cDNA equivalent to 25 ng of RNA were used per reaction, to which 15 μl of reaction mix (10 μl master mix precisionPLUS from Primerdesign, 4 μl H_2_O, 1 μl gene specific primers from Primerdesign or Sigma-Aldrich) were added. For each gene of interest, a water sample was loaded as negative control, containing 5 μl H_2_O and 15 μl reaction mix. For each sample, two technical replicates were included.

### geNorm analysis

geNorm analysis was performed using a commercially available primer kit for mouse and a custom-made primer kit for naked mole-rat, both by Primerdesign. For both analyses, 12 housekeeping gene-specific primer pairs were used and the expression of the genes was determined in two biological samples from each tissue using qPCR as described above. The geNorm software (part of qbase+, https://www.qbaseplus.com) calculated the pair-wise expression ratio of all genes, the variation of which corresponds to expression stability, resulting in a ranking of the genes based upon a gene stability measure (M). geNorm calculates how many housekeeping genes should be used for normalization by calculating a normalization factor and pairwise variation (V), which asks how the addition of the next best housekeeping genes impacts the stability of the normalization.

### ΔCT analysis

For analysis of *ASIC* expression, the ΔCT (threshold cycle) method was used [39]. CT values were normalized by subtracting the geometrical mean of the reference CT values from the sample CT value (CT_sample_ – CT_ref_ = ΔCT). The efficiency (E) was then used to determine the normalized relative abundance of transcripts by calculating E^-ΔCT^ and this value was plotted on a log_10_ axis to visualize the exponential nature of the fold change between samples/transcripts. Statistical analysis within tissues and between species has not been conducted because although primer efficiencies are comparable throughout, conditions between reactions and tissues are too different to allow for such a comparison.

## Results

### RNA integrity

**Fig. 1:**
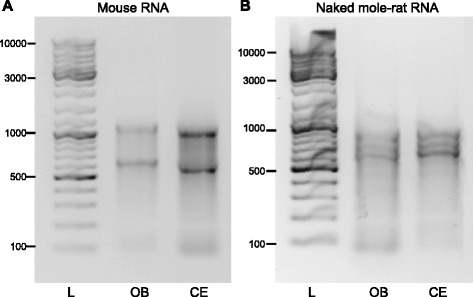
RNA samples on a 1% agarose gel after DNaseI digest and clean up. **a** Mouse RNA from olfactory bulb (OB) and cerebellum (CE), the DNA ladder (L) marks DNA sizes from 100bp to 10,000bp. **b** Naked mole-rat RNA from OB and CE

As an estimate for RNA purity and integrity, spectroscopy values and agarose gel electrophoresis were used. Following spectroscopy analysis, and only samples with a A260/A280 ratio >1.8 were used, which is an indication that they contained little to no contamination [40]. Ribosomal RNA is the most abundant and as expected was visible in the samples after DNaseI treatment and clean-up. Electrophoresis of mouse samples showed 28S and 18S ribosomal subunits (Fig. 1a), whereas naked mole-rat samples showed three bands as expected due to cleavage of the 28S subunit (Fig. 1b) [38].

### geNorm

**Fig. 2:**
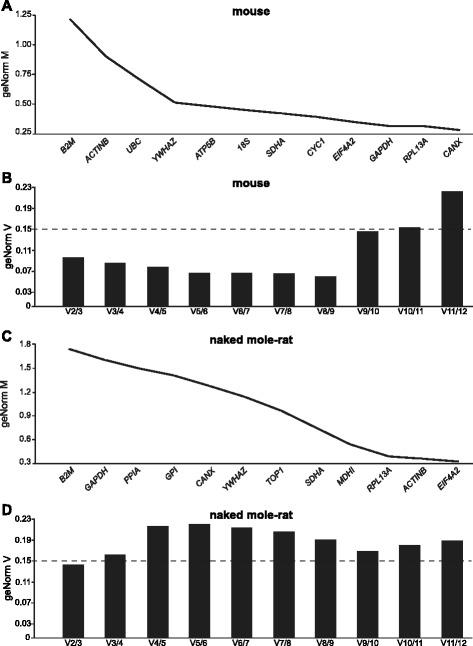
Ranking of housekeeping genes based on their expression stability between tissue samples (2 biological replicates per tissue), from low stability (*left*) to high stability (*right*). **a** Mouse geNorm experiment showing *CANX* and *RPL13A* to be the most stably expressed transcripts; M represents variation between samples based on pairwise expression ratio, a lower value means lower variability. **b** Mouse data showing the optimal number of reference genes for an experiment using samples from all tissues analyzed; V indicates if addition of more housekeeping genes, starting with the two most stable genes from plot 1, increases or decreases variation (a value below 0.15 is recommended). **c** Naked mole-rat geNorm experiment (2 biological replicates per tissue), *EIF4A2* and *β-ACTIN* were the most stably expressed transcripts. **d** Naked mole-rat data showing the optimal number of reference genes for an experiment using samples from all tissues analyzed

To find a combination of housekeeping genes that was most stably expressed between all sampled tissues, a geNorm analysis was performed. This analysis resulted in two plots, the first of which shows a ranking of all genes tested in order of expression stability (M) between tissues, with less variation (i.e. higher stability) resulting in a lower M value (Fig. 2a and c). The second plot shows pairwise V numbers, a value that expresses how reliable normalization is by using two housekeeping genes rather than three (V2/3) and how this reliability changes by adding more genes one by one (V3/4, V4/5, and so on, Fig. 2b and d). In both species, a reliable normalization could be achieved using the two most stably expressed housekeeping genes, which were *RPL13A* (ribosomal protein L13A) and *CANX* (calnexin) in mouse (Fig. 2a) and *β-ACTIN* and *EIF4A* (eukaryotic initiation factor 4a) in naked mole-rat (Fig. 2c). In the naked mole-rat tissues, only three genes were stably expressed in all tissues (Fig. 2c), and using more than two housekeeping genes for normalization would have added noise to the results (Fig. 2d) and thus for both mouse and naked mole-rat we used two housekeeping genes for normalization.

### qPCR

**Fig. 3:**
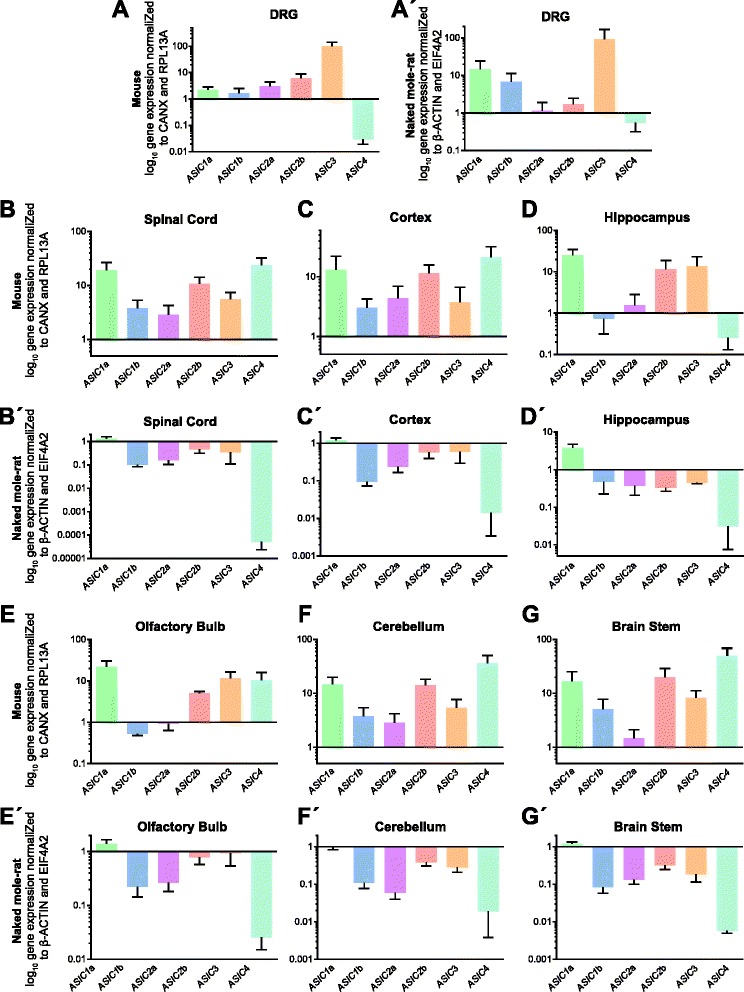
Expression *ASIC* transcripts in mouse and naked mole-rat tissues. Plots show mean and standard error of the mean (SEM) of log_10_ of *ASIC* transcripts normalized to the *CANX* and *RPL13A* for mouse tissues and *ACTINB* and *EIF4A2* for naked mole-rat tissues. **a** and **a**’ DRG (mouse, n = 3, naked mole-rat, n = 4), **b** and **b**’ spinal cord (mouse, n = 7, naked mole-rat, n = 3-4), **c** and **c**’ cortex (mouse, n = 4-5, naked mole-rat, n = 4), **d** and **d**’ hippocampus (mouse, n = 3, naked mole-rat, n = 3-4), **e** and **e**’ olfactory bulb (mouse, n = 6-8, naked mole-rat, n = 3-4), **f** and **f**’ cerebellum (mouse, n = 7, naked mole-rat, n = 4), and **g** and **g**’ brain stem (mouse, n = 6, naked mole-rat, n = 3-4). n = number of biological replicates

Relative expression fold change for different *ASIC* transcripts was determined using the ΔCT method (see Methods) in both mouse and naked mole-rat. In the mouse, all *ASIC*s were expressed in all tissues analyzed, but at distinctly different levels (Fig. 3). *ASIC3* was expressed in all tissues analyzed at comparable levels apart from in the DRG where *ASIC3* levels were approximately 10-fold higher than in all other tissues (Fig. 3a). In the spinal cord and across most brain regions, *ASIC3* was expressed at a similar level to *ASIC1a* and *ASIC2b* with *ASIC1b* and *ASIC2a* showing consistently lower expression (Fig. 3b – g). Thus in the mouse brain *ASIC* expression can be split into two groups, high expression: *ASIC1a*, *ASIC2b* and *ASIC3*, and low expression: *ASIC1b* and *ASIC2a*. With the exception of DRG and the hippocampus, where *ASIC4* showed the lowest level of expression, *ASIC4* was frequently the most highly expressed ASIC (Fig. 3a – g).

In naked mole-rat tissues, a similar pattern was observed for *ASIC* DRG expression as in mouse, *ASIC3* was the most highly expressed transcript and *ASIC4* the lowliest (Fig. 3a’). Similarly, as in mouse tissues, in naked mole-rat non-DRG tissue, expression could be grouped into a high expression group (*ASIC1a*, *ASIC2b* and *ASIC3*) and a low expression group (*ASIC1b* and *ASIC2a*) (Fig. 3b’ – g’). However, by contrast with the mouse expression profile, *ASIC4* showed the lowest level of expression in all tissues (Fig. 3a’ – g’).

## Discussion

### geNorm analysis identified optimal combination of housekeeping genes

A geNorm analysis was performed to identify the most stably expressed housekeeping genes. The gene stability value (M) is based on the assumption that the expression ratio between two control genes should be the same in all samples, therefore increasing variation in ratio means decreasing expression stability. Average M-values are usually in the range of 0.5–1 when using heterogeneous tissue samples, and values >1 indicate low stability [41, 42]. The geNorm algorithm ranked the twelve control genes according to their pairwise expression stability in five brain regions, spinal cord and DRG neurons. Studies have found that using two older programs, BestKeeper or NormFinder, which are based on the same paradigm, but use slightly different algorithms, results in significantly similar gene rankings, emphasizing the validity of the results using geNorm [43].

Analysis of human and mouse qPCR studies in the literature found that the most frequently used housekeeping genes were *GAPDH*, *18S* and *β-ACTIN*, however, evidence suggests that they might not be appropriate for all tissues and conditions, especially during development, and therefore it is important to determine the best housekeeping gene combination at the start of a qPCR study [44–47]. Moreover, no study has investigated optimal housekeeping genes for experiments using naked mole-rat tissue. As per geNorm guidelines, the optimal number of reference genes is reached when the V (pairwise variation) value falls below 0.15 [42]. In both mouse and naked mole-rat, using two reference genes for normalization fulfilled this criterion.

The stability analysis showed many similarities, but also some incongruence between the two species. It should firstly be noted that although rodents, mice and naked mole-rats belong to two different Rodentia suborders, Myomorpha and Hystricomorpha respectively and thus differences in gene stability are perhaps to be expected. Moreover, age is also a complicating factor because although adult mice (6-7-month old) were used, it is not entirely clear how the developmental stage relates to 6-month old naked mole-rat, which can live for ~30 years, although at 4-months of age the naked mole-rat brain is already 90% of the adult size [48]. The development of the eusocial naked mole-rat is somewhat different because animals do not sexually mature unless they are part of the breeding pair or are removed from the influence of the breeding female [49]. However, the animals used in this study can be considered adults at this age, because they can mature and reproduce if separated from the colony.

Although a previous study has identified β-2-microglobulin (*B2M*) as being the most stably expressed transcript in different mouse brain regions when comparing neonatal and adult tissue [40], we found *B2M* to be the most variable gene in both species (Fig. 2a and c), indicating that although perhaps useful for comparing developmental stages, *B2M* should not be used for normalization of qPCR experiments using adult mouse and naked mole-rat brain tissues. Overall, *RPL13A* and *EIF4A2* were within the four most stable genes in both species and therefore could be used for future inter-species comparisons.

Bruckert et al. tested the expression of nine commonly used reference genes in four mouse brain regions, including some of the same structures examined in our study (cortex, cerebellum and hippocampus) at different stages of development and found that at least two reference genes were needed for good normalization [50]. Interestingly, in three of the tested tissues (cortex, hippocampus, striatum), *RPL13A* was the most stable reference gene [50], and *RPL13A* was found to be a robust housekeeping gene in mouse samples in this study, too (Fig. 2a).

Although widely used as a reference housekeeping genes, Kouadjo et al. found that glyceraldehyde-3-phosphate dehydrogenase (*GAPDH*) and *β-ACTIN* can have significantly different expression between different tissues and may not be reliable in studies using very diverse tissue types [51]. Here, we find that *GAPDH* is stably expressed in mouse tissues, but not in naked mole-rat tissues, whereas the trend is reversed for *β-ACTIN* such that it is the second most stably expressed in gene in naked mole-rat tissue, but the second most unstable in mouse tissue (Fig. 2a and c). These results clearly indicate the variability of these two genes between species and tissues and demonstrate the importance of conducting gene stability analysis before choosing appropriate housekeeping genes. Interestingly, *β-ACTIN* is under positive selection in the blind mole-rat *Spalax ehrenbergi* and the resulting variation in this highly conserved gene might make the protein more resistant to oxidation [52]. It can thus be speculated that expression is also affected, which introduces the possibility that *β-ACTIN* expression is more stable in the blind mole-rat brain than in the brains of other rodents and considering the overlapping phenotype of the blind mole-rat with the naked mole-rat [22, 53] a similar positive selection and expression stability may account for *β-ACTIN* being the second most stably expressed in gene in naked mole-rat tissue.

*CANX* has been previously described as a stable reference gene between germ and somatic cells of both sexes in mice when using fetal cells [54] and a further geNorm study using eight genes on the liver tissue from the non-model mouse species *Apodemos flavicollis* (yellow-necked mouse) found *CANX* to be consistently within the three most stable genes [43]. In the current geNorm study, we demonstrate that *CANX* was the most stably expressed gene between mouse tissues, but this was not the case for naked mole-rat tissues (Fig. 2a and c). By contrast, Axtner et al*.* found ubiquitin C (*UBC*), ribosomal protein L13A (*RPL13A*) and *β-ACTIN* to be the least stable genes they examined [43], whereas *RPL13A* and *β-ACTIN* were two of the three most stably expressed genes in naked mole-rat samples (Fig. 2c) and *RPL13A* the second most stable mouse housekeeping gene, however, in agreement with Axtner et al*.*, *β-ACTIN* and *UBC* were two of the three least stable genes in mouse tissues (Fig 2a). Although some studies have warned that *β-ACTIN* is not always an appropriate gene to use for normalization because its expression can be affected by treatments and age, a majority of studies analyzed by Chapman et al. found *β-ACTIN* to be among the most stable genes in the brain, especially in the hippocampus, cortex, basal ganglia, mesencephalon, but also whole brain samples [46]. It is therefore not unusual that *β-ACTIN* was one of the two most stable reference genes in the naked mole-rat brain. The huge variation in stability of housekeeping genes between different studies clearly shows the importance of performing a geNorm (or similar) analysis at the start of each study, using the same primers and samples that will be used for subsequent experiments.

In summary, we determined that *RPL13A* and *CANX* were the most stable housekeeping genes in mouse tissues (3rd and 11th in naked mole-rat) and that *ACTINB* and *EIF4A2* were the most stable housekeeping genes in naked mole-rat tissues (11th and 4th in mouse).

### qPCR expression confirmed ASIC expression in the nervous system

Previous studies of ASIC expression relied heavily on either RNA-oligonucleotide-based (such as in situ hybridization and Northern Blot) or on antibody-based (immunohistochemistry, Western Blot) techniques. However, many studies either failed to differentiate between splice isoforms or did not determine the expression of every ASIC subunit (Table 4).

**Table 4:** ASIC expression in the nervous system

	DRG	SP	CO	HC	OL	CE	BS	Whole brain
ASIC1	NB (r[28])	NB (r[28])	ISH (m[27], r[59]) NB (r[28]) IHC (m[7])	ISH (m[27, 25], r[59]) IHC (m[7]) NB (r[28], h[25])	ISH (m[27, 25], r[59]), IHC (m[7])	ISH (m[27, 25], IHC (m[7]), NB (r[28])	NB (r[28])	NB (r[28]), ISH (m[25])
ASIC1a	qPCR (h[31, 55], m[14, 55], nmr[14])	qPCR (h[31], m[33]						qPCR (h[31, 55], m[55])
ASIC1b	qPCR (m[14, 55], nmr[14])							qPCR (m[55])
ASIC2	NB (r[36])	NB (r[36])	ISH (r[26, 36, 59])	ISH (m[25], r[26, 36, 59]) NB (h[25]) EP (m[11])	ISH (m[25], r[26, 36, 59])	ISH (m[25], r[26, 36] NB (r[36])	NB (r[36])	NB (r[36]) ISH (m[25])
ASIC2a	qPCR (m[14], nmr[14])	qPCR (m[33])						qPCR (h[31])
ASIC2b	qPCR (m[14], nmr[14])	qPCR (m[33])						qPCR (h[31])
ASIC3	RT (r[35]), NB (r[36]), qPCR (h[31], r[31], m[14], nmr[14]) WB (r[35])	qPCR (h[31], m[33])	RT (r[35]), WB (r[35])	RT (r[35]), WB (r[35])		ISH (r[59])		qPCR (h[31]),
ASIC4		NB (r[36]) ISH (r[36])	ISH (r[36]) RT (r[37])	ISH (r[36])		NB (r[36]) RT (r[37])	NB (r[36]) RT (r[37])	NB (r[36, 37])

Different techniques have been previously used to determine ASIC expression in the nervous system tissues used in this study. Abbreviations used in Table 4 are as follows *BS* brain stem, *CE* cerebellum, *CO* cortex, *DRG* dorsal root ganglia, *EP* electrophysiology, *HC* hippocampus, *IHC* immunohistochemistry, *ISH* in situ hybridisation, *NB* Northern blot, *OL* olfactory bulb, *qPCR* quantitative real-time PCR, *RT* reverse-transcriptase PCR, *WB* western blot. Species: *r* rat, *h* human, *m* mouse, *nmr* naked mole rat. Studies listed for ASIC1 and ASIC2 did not differentiate between splice isoforms

We have previously shown that *ASIC3* is most abundant in mouse DRG neurons, *ASIC1b*, *ASIC2a* and *ASIC2b* having similar levels and *ASIC1a* being least abundant (*ASIC4* was not investigated) [14]. Here we find that *ASIC3* is most abundant, but that *ASIC1b* rather than *ASIC1a* is least abundant, although both *ASIC2a* and *ASIC2b* have higher expression in accordance with our earlier study (Fig. 3a). However, it should be noted that our previous study only used one housekeeping gene for normalization (*HPRT1,* hypoxanthine phosphoribosyltransferase 1). A comparison of *ASIC1a* and *ASIC1b* expression in human DRG also found that *ASIC1b* was higher in DRG neurons than *ASIC1a*, but again a single reference gene was used (*GAPDH*) [55]. We also find that *ASIC4* is the least abundant ASIC in DRG, which is in accordance with previous data showing that *ASIC4* is sparsely expressed in DRG [8, 36, 37].

In accordance with previous accounts, *ASIC1a*, *ASIC1b*, *ASIC2a* and *ASIC2b* were expressed abundantly in the brain and spinal cord of mice [26, 27, 56]. *ASIC1b* expression was consistently lower than *ASIC1a*, which might explain why it was previously thought that this subunit was confined to DRG [28]. In a qPCR study comparing ASIC expression in the mouse spinal cord, *ASIC2a* was most highly expressed followed by *ASIC1a*, while the expression of *ASIC3* was rather low [33], whereas we find that *ASIC2a* is the lowest of the these transcripts (Fig. 3b). However, it is difficult to compare our results with those of Baron et al*.* because no error bars are provided in their data and their study only used a single reference gene, 18S, which for reasons discussed previously may not be the most appropriate reference gene to use.

With regards to levels of *ASIC* transcripts in the brain, *ASIC1a* is consistently highly expressed (Fig. 3c – g), which is to be expected considering the importance of ASIC1a in neuronal physiology and animal behavior [5–7, 11, 12, 57]. *ASIC1b* expression is along with that of *ASIC2a* commonly the lowliest expressed across different brain regions, whereas *ASIC2b* shows similar levels to *ASIC1a*. Previous reports have reached little consensus about *ASIC3* expression in the brain, but here we demonstrate that *ASIC3* is strongly expressed in all tissues investigated, and a RT-PCR study by Meng et al also showed *ASIC3* transcripts present in different parts of the brain, in addition to DRGs [35]. Lastly, as others have shown with a variety of techniques [8, 36, 37], *ASIC4* was highly expressed in all brain regions analyzed as well as the spinal cord, where it was more abundant than other ASICs.

To date, nothing is known about *ASIC* expression in the naked mole-rat brain. However, considering the variable roles of ASICs in neuronal physiology and behavior, it is important for ASIC expression to be understood in the naked mole-rat as differential ASIC function and/or expression could alter neuronal acid sensitivity. This is of particular importance in a species that is known to be adapted to its hypercapnic, subterranean environment, such that a variation in the amino acid sequence of NaV1.7 confers behavioral acid-insensitivity in naked mole-rats [14, 19]. We have previously shown using qPCR that as in mouse *ASIC3* is most abundantly expressed in DRG, followed by *ASIC1a* and *ASIC1b*, with *ASIC2a* and *ASIC2b* being more lowly expressed [14] and in this study we observed an almost identical trend, but with *ASIC2a* actually showing slightly lower expression that *ASIC2b* (Fig. 3a’); we also show for the first time that, as in mouse, *ASIC4* is the least abundant ASIC transcript.

**Fig. 4:**
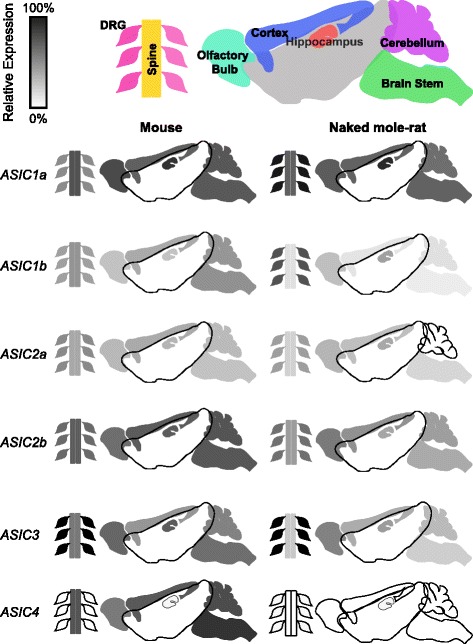
Summary of *ASIC* expression determined by qPCR in the olfactory bulb, cerebellum, brain stem, cortex, hippocampus, spinal cord and DRG neurons. Shades of grey indicate the level of *ASIC* expression in each tissues for mouse and naked mole-rat. For each species, the sample with the highest expression (in both cases *ASIC3* in the DRG) was used as a reference (100% expression) and the relative expression in other tissues was calculated from this for each species independently

When comparing the central nervous system between mouse and naked mole-rat, trends were generally similar, i.e. *ASIC1a* was the frequently the most highly expressed and *ASIC1b* and *ASIC2a* showing much lower abundance levels (Fig. 3b’ – g’). However, the greatest difference observed is that whereas *ASIC4* is highly abundant in all mouse central nervous system tissues tested here apart from the hippocampus, in naked mole-rat central nervous system tissues *ASIC4* is consistently the least abundant (Fig. 3b’ – g’). It has been suggested that *ASIC4* plays a modulatory role and can interact with polyubiquitin to downregulate other ASICs [58] and recent evidence suggests that it can counteract the activity of ASIC1a in the brain to modulate fear and anxiety behavior [8]. Thus, it can be speculated that one of these functions, or another as of yet unknown function of the subunit, has been lost or made redundant in naked mole-rat, although the physiological properties of naked mole-rat ASIC4 are currently unknown. A summary of *ASIC* expression in mouse and naked mole-rat tissue is illustrated in Fig. 4 and can be compared to that published by others using a variety of techniques in Table 4.

In conclusion we have conducted a geNorm analysis of mouse and naked mole-rat nervous system tissues to identify appropriate housekeeping genes for normalization. We have then used a robust and uniform strategy to determine *ASIC* transcript expression in both the mouse and the naked mole-rat. Our results will benefit those who aim to perform qPCR studies in the naked mole-rat, which is becoming an ever more widely used laboratory species, as well as providing further insight into the role of ASICs.

## Abbreviations

18S: 18s ribosomal subunit
ASIC: Acid-sensing ion channel
ATP5b: ATP synthase, H^+^ transporting, mitochondrial F1 complex, beta polypeptide
B2M: Beta-2-microglobulin
BMR: Blind mole-rat (*Spalax galili*)
bp: Base pairs
BS: Brain stem
cDNA: Complementary DNA
CE: Cerebellum
CNX: Connexin
CO: Cortex
CT: Cycle threshold
CYC1: Cytochrome C1
dATP: Deoxyadenosine triphosphate
dCTP: Deoxycytidine triphosphate
dGTP: Deoxy guanosine triphosphate
DNA: Deoxyribonucleic acid
DRG: Dorsal root ganglia
dTTP: Deoxy thymidine triphosphate
E: Efficiency
EIF4A2: Eukaryotic initiation factor 4A2
fw: Forward
g: Gram(s)
GAPDH: Glyceraldehyde 3-phosphate dehydrogenase
GPI: Glucose-6-phosphate isomerase
HC: Hippocampus
L: Ladder
M: geNorm gene-stability measure
mRNA: Messenger RNA
n: Number of biological replicates
NaV: Voltage-gated sodium channel
OB: Olfactory bulb
PCR: Polymerase chain reaction
RPL13A: Ribosomal protein L13a
rRNA: Ribosomal RNA
RT: Room temperature
RT-PCR: Reverse-transcriptase PCR
rv: Reverse
SDHA: Succinate dehydrogenase complex, subunit A
SEM: Standard error of the mean
SP: Spinal cord
TM: Melting temperature
TRPV1: Transient receptor potential cation channel subfamily V member 1
UBC: Ubiquitin C
V: geNorm pairwise variation measure
YWHAZ: Tyrosine 3-monooxygenase/tryptophan 5-monooxygenase activation protein zeta
